# Accuracy of Computed Tomography Angiography for Diagnosing Extracranial Mural Lesions in Patients with Acute Internal Carotid Artery Occlusion: Correlation with Digital Subtraction Angiography

**DOI:** 10.3390/jpm13071169

**Published:** 2023-07-21

**Authors:** Miriam Fernández-Gómez, Félix Gallo-Pineda, Carlos Hidalgo-Barranco, Gracia Castro-Luna, Patricia Martínez-Sánchez

**Affiliations:** 1 Interventional Neuroradiology, Torrecardenas University Hospital, University of Almería, 04009 Almería, Spain; miriamfgomez89@gmail.com (M.F.-G.); gallopineda@gmail.com (F.G.-P.); carlos_h87@hotmail.com (C.H.-B.); 2 Faculty of Health Sciences, University of Almería, 04120 Almería, Spain; 3 Stroke Centre, Department of Neurology, Torrecardenas University Hospital, University of Almería, 04009 Almería, Spain; patrindalo@hotmail.com

**Keywords:** internal carotid artery, occlusion, angiography, etiology, dissection, tandem, thromboembolism

## Abstract

Extracranial carotid mural lesions (CML), caused by atherosclerosis or dissection, are frequently observed in acute internal carotid artery (ICA) occlusion, often requiring angioplasty or stenting. This study aimed to assess the diagnostic accuracy of computed tomography angiography (CTA) in differentiating extracranial CML from thromboembolic etiology in acute ICA occlusion in patients eligible for endovascular treatment. Two neuroradiologists retrospectively studied patients with apparent extracranial ICA occlusion on CTA. Patients were divided into two groups: thromboembolism and CML, based on findings from CTA and digital subtraction angiography (DSA). CTA sensitivity and specificity were calculated using DSA as the gold standard. Occlusive patterns and cervical segment widening were evaluated for atherosclerosis, dissection, and thromboembolism etiologies. CTA had a sensitivity of 84.91% (74.32–95.49%) and a specificity of 95.12% (87.31–100%) in detecting extracranial CML. Atherosclerosis was the most common cause, distinguishable with high accuracy using CTA (*p* < 0.001). No significant differences were found in occlusive patterns between dissection and thromboembolism (*p* = 0.568). Cervical segment widening was only observed in dissection cases due to mural hematoma. Conclusions: CTA accurately differentiates extracranial CML from thromboembolic etiology in acute ICA occlusion. The pattern of the occlusion and the artery widening help to establish the location and the etiology of the occlusion.

## 1. Introduction

In recent years, endovascular therapy (ET) has changed the treatment of acute ischemic stroke due to large vessel occlusion, significantly reducing mortality and morbidity [1,2,3,4]. This fact has been demonstrated in the results of recent clinical trials, which have led to a change in the prognosis of these patients [1,2,3,4].

Acute ischemic stroke resulting from internal carotid artery (ICA) occlusion is associated with a poor prognosis, often leading to severe stroke and extensive cerebral infarction [5,6,7,8]. Acute ICA occlusions account for 8–15% of patients with stroke caused by large vessel occlusion. The ICA distally divides into the middle cerebral artery and the anterior cerebral artery, making it the main artery supplying the cerebral parenchyma [9]. In patients presenting with anatomical variations, such as the hypoplasia or aplasia of segment A1 in the contralateral anterior cerebral artery, or in the case of the fetal origin of the ipsilateral posterior cerebral artery, the affected vascular territory may exhibit further enlargement, carrying prognostic implications [5]. Furthermore, the status of collaterals and vascular compensation significantly impacts the ultimate prognosis for these individuals [10].

The location of the acute ICA occlusion can be categorized into intracranial, extracranial, or tandem, in the case of concomitant intracranial and extracranial occlusions [6,11,12]. Given the typical dimensions of the ICA, occlusion in this region can commonly be attributed to a large embolus, primarily originating from the heart [5]. However, it can also be associated with the presence of carotid mural lesions (CML), such as atherosclerosis or dissection [5,9,11]. Atherosclerosis is the leading cause of extracranial or tandem ICA occlusions, whereas thromboembolism more often causes intracranial occlusion [5,11]. Nevertheless, oversized thromboembolisms can affect the cervical segment or the origin of the ICA [13,14]. Dissections, on the other hand, predominantly impact the distal cervical segment of the ICA near the base of the skull [5,11].

Non-contrast computed tomography (NCCT) and computed tomography angiography (CTA) are commonly performed before ET to determine the location and the etiology of the arterial occlusion [1,2,3,4,15,16,17,18,19]. Identifying the exact location of the occlusion, intracranial or extracranial, and the presence of extracranial CML resulting from atherosclerosis or dissection is crucial in selecting appropriate devices and revascularization techniques [9,20,21,22,23,24]. The pattern of the ICA occlusion on CTA has been studied with the aim of predicting the location or the etiology before ET [25,26]. However, different terms were used to refer to the same morphology, with divergent outcomes among them [23,24,25,26]. Artery widening is frequently associated with mural hematoma, being a specific radiological sign of dissection [5,11].

Our study aimed to evaluate the diagnostic capabilities of CTA in recognizing extracranial CML in patients with acute ICA occlusion before ET. Furthermore, we assessed the radiological signs which play a crucial role in distinguishing between different etiologies, including the pattern of the occlusion and the arterial widening.

## 2. Materials and Methods

### 2.1. Study Design

This study was a single-center retrospective analysis of data collected from patients treated with ET due to acute ischemic stroke caused by large vessel occlusion between October 2017 and May 2023.

The study was conducted at the reference center for ET in Southern Europe in a geographic area with a population of approximately 740,000 inhabitants. During the specified time period, a total of 464 mechanical thrombectomies were performed to address the occlusion of large vessels, any level of occlusion, in both the anterior and posterior circulation. Notably, 130 procedures were conducted in the most recent year (2022).

### 2.2. Patient Selection

The inclusion criteria were the apparent extracranial ICA occlusion on CTA in patients with acute extracranial or intracranial ICA occlusion confirmed on DSA. The exclusion criteria were the presence of a different occluded artery and the non-availability or non-valuable NCCT, CTA, or digital subtraction angiography (DSA).

This study was approved by the local ethics committee (71/2022) and performed according to the ethical standards laid down in the Declaration of Helsinki.

### 2.3. Image Acquisition Protocol

All patients with stroke suspected of large vessel occlusion in our center underwent NCCT and CTA following the recommendations of the American Heart/Stroke Association [15].

First, NCCT scans were acquired from the skull base to the vertex to rule out hemorrhagic or other non-vascular causes of stroke. Second, CTA was performed to detect the location and the etiology of the occlusion. For this technique, 80 cubic centimeters of intravenous contrast (Ioversol 350 milligram/cubic centimeter) were administered through an injection pump at a flow rate of 4 milligrams per second, followed by a saline flush. Subsequently, CTA scans were obtained from the aortic arch to the vertex using contrast bolus monitoring, with ROI (region of interest) utilized for image analysis. Delayed phases were acquired in all patients, but they were not the focus of this study.

NCCT and CTA were both acquired using a 64-slice CT scanner at the mechanical thrombectomy reference center. Image post-processing consisted of the multiplanar reconstruction to obtain axial, coronal, and sagittal planes. Additionally, maximum intensity projection was performed with a slab thickness of either five or ten millimeters.

DSA was performed in an Artis Zee biplane (Siemens) before ET to detect the location and the etiology of the occlusion. Sagittal, coronal, and/or oblique cerebral angiographies were sequentially acquired with manual iodinated contrast administration through a catheter located in the common carotid artery and/or ICA. DSA served as the gold standard to establish the definitive location and etiology of the occlusion, as well as to identify the presence of CML [19,27].

### 2.4. CTA Analysis

Two interventional neuroradiologists with six and five years of experience in vascular diseases, MFG and FGP, respectively, independently conducted a blinded analysis of the CTA of the patients with apparent extracranial ICA occlusion. The location of the extracranial ICA occlusion was categorized into the origin (within the first two centimeters) and the cervical segment occlusion.

Later, the patients were classified into three etiologies (thromboembolism, atherosclerosis, and dissection) based on CTA radiological signs described in previous research.

Thromboembolism: This category included cases with apparent extracranial ICA occlusion, lacking calcifications, atherosclerotic plaque, or wall hematoma at the site of occlusion [28,29].

Atherosclerosis: This classification was assigned to cases with severe calcifications or plaque observed at the occlusion site [11,14,28].

Dissection: Cases were classified as dissections when there was evidence of a wall hematoma (the widening of the artery), progressive stenosis, or an intimal flap [29,30,31].

Atherosclerosis and dissection cases were then included as instances of extracranial occlusion caused by CML. In cases of discordance in interpretation or classification, a consensus was reached between the two neuroradiologists.

Furthermore, the morphology of the extracranial ICA occlusion on CTA was evaluated and categorized into two forms: well-defined and flame-shaped. The latter was defined as a progressive decline in contrast due to a stagnant column of un-opacified blood proximal to the site of occlusion [23,26,32]. The presence of artery widening on CTA was assessed in all cases.

### 2.5. DSA Analysis

Two neuroradiologists (MFG and FGP) independently conducted a blinded assessment of the DSA images to ascertain the cause and the location of the occlusions in the ICA. First, the location of the ICA occlusion was further categorized into origin, cervical, and intracranial. Consequently, consistent with the analysis performed on CTA, the patients were classified into three distinct categories: thromboembolism, atherosclerosis, and dissection, as per the following criteria.

Thromboembolism: This diagnosis was assigned when no stenotic lesions were observed during ET.

Atherosclerosis: Occlusions attributable to atherosclerosis were determined by the presence of circumferential involvement of the vessel wall and characterized by calcifications or atheromatous plaque that impeded the easy passage of a guidewire [11].

Dissection: Cases were confirmed as dissections when an irregular vascular wall exhibited a conical contrast, leading to an occlusion above the level of the carotid bulb, which was not readily traversable with the guidewire [11].

Extracranial occlusions resulting from atherosclerosis and dissection were assigned to the CML group, while the remaining patients were classified under the thromboembolism (TE) group.

### 2.6. Demographic Variables, Comorbidities, and Clinical Severity

The demographic variables analyzed in this study comprised the age and sex of the patients, with specific attention given to the proportion of male participants. Additionally, a comprehensive examination of cardiovascular risk factors was conducted, encompassing the presence of arterial hypertension, dyslipidemia, diabetes mellitus, history of atrial fibrillation, de novo atrial fibrillation diagnosis, history of ischemic heart disease and stroke, as well as tobacco and alcohol consumption. Furthermore, the clinical severity of the patients was evaluated using the National Institutes of Health Stroke Scale (NIHSS). These variables were compared between the two groups, TE and CML.

### 2.7. Statistical Analysis

IBM SPSS Statistics^®^ v28 and Epidat 3.1^®^ programs were used for the statistical analysis. The Kolmogorov–Smirnov normality test was performed for the different variables studied. Student’s *t*-test was used for the comparison of means between normal quantitative and qualitative variables. We employed Fisher’s test to compare the means between two non-normal qualitative variables. The Mann–Whitney U-test allowed us to compare the means between qualitative and non-normal quantitative variables.

In addition, CTA sensitivity and specificity were calculated for the detection of extracranial CML in patients with acute ICA occlusion. DSA was the gold standard.

## 3. Results

Between October 2017 and May 2023, 106 patients were treated by ET for acute ICA occlusion. Ninety-four of them, who exhibited apparent extracranial ICA occlusion on CTA, were included in the study. Following the analysis of the DSA results, the 94 patients were categorized into two groups: the TE group (*n* = 42, 44.7%) and the CML group (*n* = 52, 55.3%).

Regarding demographic and comorbidity data, statistically significant differences were observed between the two groups in various variables. Specifically, the TE group had a higher mean age (74 vs. 66, *p* = 0.008) and a greater proportion of patients with a history of atrial fibrillation (33.3% vs. 5.8%, *p* < 0.001).

Conversely, the CML group had a higher ratio of male patients (82.3% vs. 42.8%, *p* < 0.001) and smokers (61.5% vs. 19%, *p* < 0.001). In terms of stroke severity, the National Institutes of Health Stroke Scale (NIHSS) score was higher in the TE group (18 vs. 15, *p* = 0.007). No other statistically significant differences were found between the groups in the remaining variables (Table 1).

**Figure 1:**
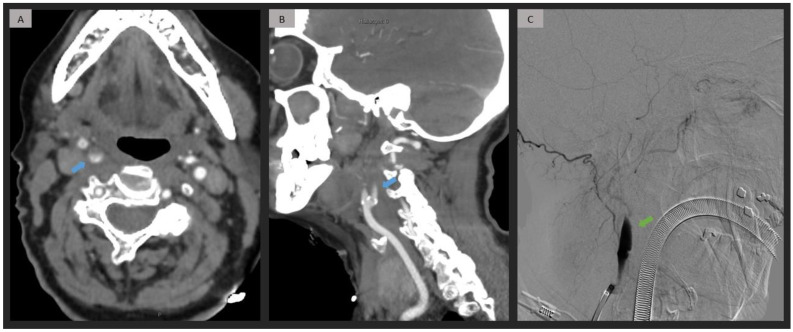
Right extracranial ICA occlusion caused by thromboembolism in a patient with a history of atrial fibrillation. CTA (**A**,**B**) shows an extracranial short progressive contrast decline (blue arrow), with calcifications in the origin of the ICA, proximal to the occlusion site. DSA (**C**) confirms a cervical ICA occlusion (green arrow) due to thromboembolism, without carotid mural lesions. ICA = internal carotid artery; CTA = computed tomography angiography; DSA = digital subtraction angiography.

**Figure 2:**
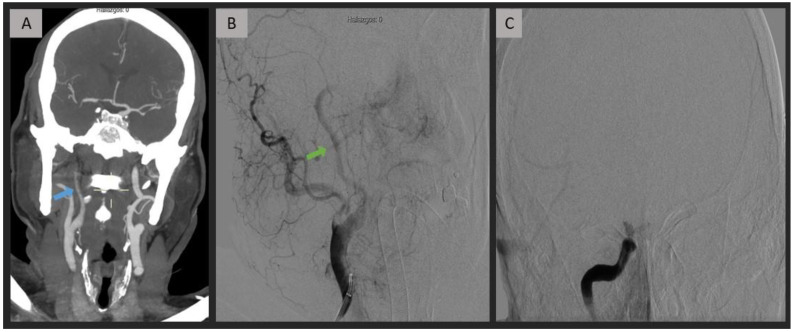
Acute intracranial ICA occlusion caused by thromboembolism. CTA (**A**) demonstrates a progressive contrast decay in the extracranial region, extending towards the distal right ICA (blue arrow), while maintaining patency of the terminal bifurcation. DSA images (**B**,**C**) confirm an isolated intracranial occlusion while indicating the patency of the extracranial segment (green arrow). ICA = internal carotid artery; CTA = computed tomography angiography; DSA = digital subtraction angiography.

Within the TE group (*n* = 42), a distinction was made between cases of extracranial true occlusion (*n* = 21, 50%) and pseudo-occlusion resulting from an intracranial location (*n* = 21, 50%) (Figure 1 and Figure 2). In cases of extracranial true occlusion attributed to thromboembolism, the clot was found in the cervical segment (*n* = 12, 28.6%) and the origin of the ICA (*n* = 9, 21.4%) (see Table 2).

**Figure 3:**
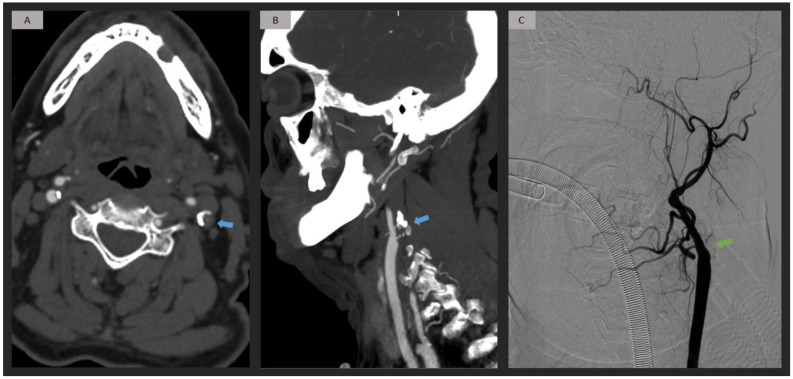
Patient with left origin ICA occlusion attributed to an atherosclerotic plaque. Axial and sagittal CTA images (**A**,**B**) exhibit a well-delimited occlusion at the origin of the ICA, resulting from an atherosclerotic plaque with calcifications (blue arrow). DSA confirms the occlusion of the ICA from its origin (green arrow). (**C**). ICA = internal carotid artery; CTA = computed tomography angiography; DSA = digital subtraction angiography.

**Figure 4:**
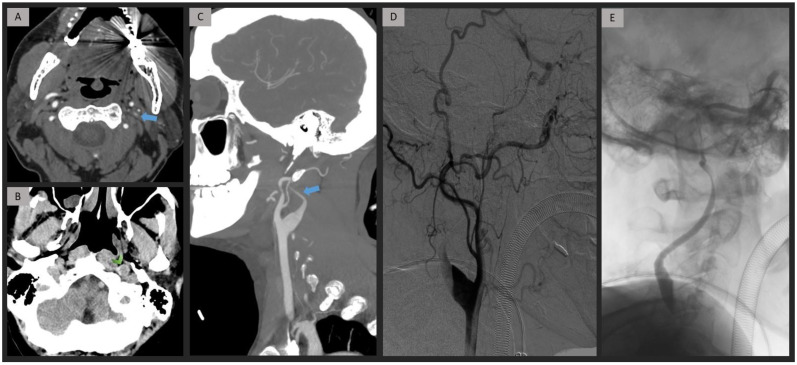
Patient presenting with left extracranial ICA occlusion caused by dissection. Axial and Sagittal-MIP CTA images (**A**,**C**) demonstrate a progressive contrast decay and stenosis of the cervical ICA, associated with an arterial widening (blue arrow). NCCT (**B**) reveals a wall hematoma in the left ICA near the skull base (green arrowhead). DSA (**D**) and fluoroscopy (**E**) confirm the diagnosis of cervical ICA dissection. ICA = internal carotid artery; NCCT = non-contrast computed tomography; MIP = maximum intensity projection; CTA = computed tomography angiography; DSA = digital subtraction angiography.

The CML group (*n* = 52) was divided into atherosclerosis (*n* = 46, 88.5%) and dissection etiologies (*n* = 6, 11.5%). Extracranial occlusion caused by atherosclerosis was predominantly observed at the origin of the ICA (*n* = 44, 95.6%) (Figure 3). Conversely, we noted that the cervical segment was the location for extracranial occlusion secondary to dissection (*n* = 6, 100%). Within the dissection etiology, a widening of the cervical segment due to mural hematoma was identified in four of six patients (66.7%) (Figure 4) (Table 2).

CTA analysis revealed significant differences in occlusion patterns between atherosclerosis and the other etiologies (thromboembolism and dissection). A well-defined pattern was more commonly observed in atherosclerosis cases (93.5% vs. 21.4% and 93.5% vs. 0%, *p* < 0.001). However, no significant differences in occlusion patterns were found when comparing the thromboembolism group to the dissection subgroup. Both groups predominantly exhibited a flame-shaped pattern, with frequencies of 78.6% and 100%, respectively (*p* = 0.568) (Table 2).

CTA sensitivity and specificity for the detection of extracranial CML in patients with apparent extracranial ICA occlusion were 84.91% (95% confidence interval: 74.32–95.49%) and 95.12% (95% confidence interval: 87.31–100%), respectively.

## 4. Discussion

The non-invasive imaging techniques used in acute ischemic stroke patients, NCCT and CTA, aim to identify the exact location and cause of the arterial occlusion. In patients eligible for ET, assessing the presence of extracranial CML in acute ICA occlusions at the occlusion site is essential for procedure planning, because these lesions may require stenting or angioplasty. However, in the absence of these CML, a thromboembolic cause should be considered. In such cases, treatment options may include mechanical thrombectomy using aspiration and stent retrievers or aspiration alone for thrombus removal [12,13]. Therefore, a correct diagnosis is necessary for careful procedure preparation and for the selection of suitable devices [12,21,33,34,35,36,37].

CML caused by atherosclerosis or dissection can induce severe cerebrovascular stroke within the territory of the ICA for several reasons. These include significant hemodynamic stenosis without compensation from the Willis polygon or occlusion and thrombosis resulting from intimal exposure to the bloodstream, leading to platelet aggregation [5]. In both instances, it is imperative to employ stenting/angioplasty procedures to restore the artery’s caliber and stabilize the plaque or intimal flap, thereby preventing its exposure to circulating blood [35,36].

One of the main objectives of our study was to evaluate the efficacy of CTA in detecting extracranial CML in cases of acute ICA occlusion. Such occlusions usually occur as a result of stenotic lesions, in which atherosclerosis represents the predominant underlying cause [35]. While atherosclerotic plaques are commonly detected at the origin of the ICA, dissection is frequently observed in the cervical segment [5,24,27]. Extracranial thromboembolic occlusion, on the other hand, may be located in the cervical segment or at the origin of the ICA [32].

The findings of our analysis of demographics and cardiovascular risk factors in the CML and TE groups align with previous studies. Specifically, patients with CML had a lower average age and encompassed higher proportion of male individuals compared to the TE group. Furthermore, the prevalence of smoking was higher in the CML group. On the other hand, the TE group had a greater proportion of patients with atrial fibrillation, which is consistent with the findings of previous studies. It is important to clarify that factors contributing to thromboembolism, beyond atrial fibrillation, were not evaluated in this study. These factors may include the cardiomyopathy of various etiologies, valvular disease, or other types of arrhythmias, including undetected paroxysmal atrial fibrillation [5].

In our study, CTA demonstrated notable efficacy in assessing the presence of extracranial CML in acute ICA occlusion. In cases of occlusion related to atherosclerosis, the identification of atherosclerotic plaques or calcific lesions typically occurs in the origin or proximal third of the cervical segment of the ICA, at the site of occlusion [11,26,27]. Regarding dissections, the prevailing characteristic is the presence of a widening arterial due to a mural hematoma, predominantly observed in the distal cervical segment proximal to the skull base [5,6,11]. In the absence of these findings, thromboembolism emerges as the most frequent etiology [11,28]. This is in agreement with the findings of our study (see Table 2).

We simplified the carotid occlusion pattern on CTA into two types: well-defined and flame-shaped occlusions. In cases of well-defined occlusion, a distinct halt in contrast opacification can be observed in the extracranial ICA on CTA, accompanied by a clearly distinguishable boundary between the patent and occluded segments. The flame-shaped morphology arises as a result of the gradual reduction in contrast enhancement caused by a stagnant column of non-opacified blood proximal to the occlusion site [23,26,32]. This occurs when the occlusion is located a certain distance away from the origin of the ICA, in the cervical or intracranial segments [11,23,26,32].

An important consideration, which has been analyzed in several previous studies, is the presence of extracranial pseudo-occlusion on CTA resulting from an isolated intracranial ICA occlusion. The prevalence of this phenomenon varies across previously published studies, ranging from 45% to 67% of the terminal occlusions of the ICA [20,23,26,38]. This finding could originate as a consequence of the absence of an arterial escape route due to the extension of the thrombus [23,38]. Our study yielded similar results, indicating that 50% of apparent extracranial ICA occlusions on CTA, attributed to thromboembolic etiology, were exclusively localized within the intracranial ICA region, as confirmed by DSA. A flame-shaped pattern is typically observed in its presentation, and in certain studies, it has been mistaken for a dissection [11,23,24]. Previously, the term “pseudoocclusion” also designated the high-grade extracranial stenosis of the ICA that impeded contrast flow [11].

Previous studies have examined the relationship between carotid occlusion patterns on CTA and the location of occlusion in patients presenting apparent extracranial ICA occlusion on CTA [26,32]. However, the terminology used to describe each type of occlusion pattern varies among studies. For example, Kim et al. investigated the association between gradual contrast decline, carotid occlusion patterns, and the total length of contrast filling in the proximal ICA to differentiate between extracranial pseudo-occlusion and true occlusion [32]. In their study, patients with extracranial pseudo-occlusion, resulting from isolated intracranial occlusion, displayed a gradual contrast decline and a beak-shaped pattern [32]. On the other hand, Prakkamakul et al. aimed to distinguish extracranial pseudo-occlusion from true occlusion by categorizing the patterns as flame-shaped, blunt/beak-shaped, and tubular-shaped, associating the beak-shaped pattern with proximal occlusions and the flame-shaped pattern with isolated intracranial ICA occlusion [26]. In the literature, the commonly utilized term is flame-shaped, which refers to the progressive decrease in contrast in the extracranial ICA on CTA, and it has been linked to both dissection and pseudo-occlusion [11,23,24,36,38].

Taking into account all of the above, the morphology of the occlusion cannot differentiate between dissection and thromboembolism. In both cases, flame-shaped is the most frequent pattern, including extracranial pseudo-occlusion [23,24,26]. However, when the occlusion occurs at the origin, the escape route effect of the external carotid artery assists in delineating the occlusion. A well-defined occlusion pattern is more likely to be related to atherosclerotic etiology (usually with an origin location) [23,26,32]. Therefore, we believe that the pattern of occlusion is not correlated with the etiology but rather with the localization of the occlusion.

While it is imperative to acknowledge the inherent limitations of this study, encompassing its retrospective nature and single-center performance, it is noteworthy to underscore that this investigation presents one of the most extensive series documented that evaluates the accuracy of CTA for the diagnosis of extracranial CML in acute ICA occlusions, while also establishing a differential diagnosis. CTA analysis by neuroradiologists has a higher sensitivity and specificity for the diagnosis of CML in acute ICA occlusions. However, in future studies, it would be worthwhile to involve a larger number of observers, including general radiologists or neurologists who lack experience in vascular diseases. These individuals are typically responsible for analyzing imaging studies in emergency departments. For the purpose of analysis, atherosclerosis and dissection were grouped together in the CML group, distinguishing them from patients with ICA occlusion resulting from thromboembolism in the TE group. It is important to note that atherosclerosis and dissection are distinct entities with different etiopathogenic mechanisms. However, their endovascular management is similar: the recanalization or restoration of the arterial wall due to the hemodynamic effect of significant stenosis or in situ thrombosis formation.

Nevertheless, future prospective multicenter studies are imperative to validating our outcomes comprehensively and extending the generalizability of the findings to a broader population.

## 5. Conclusions

CTA is useful for distinguishing between CML and thromboembolic etiology in cases of acute ICA occlusion.

The pattern of occlusion observed in CTA allows differentiation between occlusions caused by atherosclerosis, which usually occur at the origin of the ICA, and those caused by dissection or thromboembolism, which are usually located in the cervical segment. Thromboembolism and dissection show similar occlusive patterns on CTA. Arterial widening could be indicative of mural hematoma in dissection occlusions.

In the CML group, the mean age was lower compared to the TE group, while the percentage of males and smokers was higher. Conversely, in the TE group, there was a higher prevalence of atrial fibrillation than in the CML group. These findings, along with imaging results, provide valuable insights for etiological diagnosis.

## Figures and Tables

**Table 1:** Demographic variables, comorbidities, and clinical severity according between thromboembolism (TE) and carotid mural lesion (CML) groups.

	TE Group (*n*= 42)	CML Group (*n* = 52)	*p*-Value *
Male sex, *n*, (%)	18 (42.8%)	43 (82.3%)	<0.001
Age, median (IQR), years	74 (20)	66 (15)	0.008
Arterial Hypertension, *n*, (%)	28 (66.7%)	34 (65.4%)	0.658
Diabetes Mellitus, *n*, (%)	10 (23.8%)	17 (32.7%)	0.497
Dyslipidemia, *n*, (%)	13 (31%)	28 (53.8%)	0.06
Previous Atrial Fibrillation, *n*, (%)	14 (33.3%)	3 (5.8%)	<0.001
Novo Atrial Fibrillation, *n*, (%)	6 (14.3%)	2 (3.8%)	0.071
Previous CHD, *n*, (%)	5 (11.9%)	5 (9.6%)	0.740
Previous stroke, *n*, (%)	4 (9.5%)	8 (15.4%)	0.545
Smoking, *n*, (%)	8 (19%)	32 (61.5%)	<0.001
Alcohol abuse, *n*, (%)	5 (11.9%)	10 (19.2%)	0.571
NIHSS, median, (IQR)	18 (6)	15 (13)	0.007

IQR = interquartile range; CHD= coronary heart disease; NIHSS = National Institute of Health Stroke Scale; * statistical significance *p* < 0.05.

**Table 2:** Location of the occlusion and radiological signs in patients with acute ICA occlusion according to etiology.

	Etiology Confirmed on DSA		
	Thromboembolism *n* = 42	Atherosclerosis *n* = 46	Dissection *n* = 6
Location confirmed on DSA			
Origin of the ICA, *n*, (%)	9, (21.4%)	44, (95.6%)	0, (0%)
Cervical, *n*, (%)	12, (28.6%)	1, (2.1%)	6, (100%)
Isolated Intracranial (pseudo-occlusion), *n*, (%)	21, (50%)	1, (2.1%)	0, (0%)
**Extracranial occlusion pattern on CTA**			
Well-defined occlusion, *n*, (%)	9, (21.4%)	43, (93.5%)	0, (0%)
Flame-shaped pattern, *n*, (%)	33, (78.6%)	3, (6.5%)	6, (100%)
**Other radiological sign**			
Widening of the cervical segment of ICA, *n*, (%)	0, (0%)	0, (0%)	4, (66.7%)

DSA = digital subtraction angiography; CTA = computed tomography angiography; ICA = internal carotid artery.

## Data Availability

Upon request, the corresponding author can provide the data presented in this study. However, due to personal data protection, the data are not available to the public.
